# A longitudinal study on the relation between parenting and Toddler’s disruptive behavior: what is the role of Toddler’s negative emotionality and physiological stress reactivity?

**DOI:** 10.3389/fpsyg.2024.1444447

**Published:** 2024-09-09

**Authors:** Marijke Huijzer-Engbrenghof, Loes van Rijn-van Gelderen, Hannah Spencer, Christiane Wesarg-Menzel, Nicole Creasey, Esmee S. Lalihatu, Geertjan Overbeek

**Affiliations:** ^1^Preventive Youth Care, Research Institute of Child Development and Education, University of Amsterdam, Amsterdam, Netherlands; ^2^Institute of Education and Child Studies, Leiden University, Leiden, Netherlands; ^3^Institute for Psychosocial Medicine, Psychotherapy and Psychooncology, Jena University Hospital, Friedrich-Schiller University, Jena, Germany; ^4^Department of Child and Adolescent Psychiatry/Psychology, Erasmus MC, University Medical Center Rotterdam, Rotterdam, Netherlands; ^5^Department of Clinical, Educational and Health Psychology, Division of Psychology and Language Sciences, Faculty of Brain Sciences, University College, London, United Kingdom; ^6^The Generation R Study Group, Erasmus MC, University Medical Center Rotterdam, Rotterdam, Netherlands; ^7^Kohnstamm Institute, Amsterdam, Netherlands

**Keywords:** disruptive behavior, parenting, temperament, stress reactivity, longitudinal

## Abstract

Harsh and unsupportive parenting is a risk factor for the development of disruptive behavior in children. However, little is known about how children’s temperament and stress reactivity influence this relation. In a three-wave longitudinal study, we examined whether the associations between parenting practices (supportive parenting, positive discipline, and harsh discipline) and child disruptive behavior were mediated by child temperament (negative emotionality) and stress reactivity (heart rate reactivity). In 72 families (Mage child = 14.6 months), living in the Netherlands, parents reported on their parenting practices and their children’s disruptive behavior and negative emotionality. Children’s heart rate reactivity was assessed through a series of stress-inducing tasks. Results from regression-based mediation analyses with bootstrapping showed that negative emotionality and stress reactivity did not mediate the relation between parenting and disruptive behavior. The results overall demonstrate that in a group of children this age, a reinforcing dynamic between parenting, child stress and disruptive behavior is not yet firmly established.

## Introduction

As infants transition to toddlerhood, they develop more mobile and willful behavior, while still not being fully able to regulate their emotions. This can lead to behaviors that can be challenging for parents to manage and can lead to more serious disruptive problem behavior. Disruptive behavior in childhood is characterized by disobedience, defiance of authority, an angry or irritable mood state, and verbal or physical aggression toward others. While disruptive behavior is part of normative development (Scaramella and Leve, 2004) and typically decreases with age (Olson et al., 2017), if it persists or worsens over time, it can cascade into pervasive impairments in emotional, social, and academic functioning. Consequently, disruptive behavior can increase the risk of later clinical diagnoses of externalizing disorders (Caspi et al., 2020). Moreover, early-onset disruptive behavior is associated with increased risk of health problems, school drop-out, substance abuse, and delinquency, which carries substantial emotional and financial costs to the individual and society (Rivenbark et al., 2018). This spillover effect from one domain or developmental system to another features a developmental cascade that may alter the course of development [see Masten and Cicchetti (2010)]. Thus, it is important to either reduce or prevent the worsening of children’s disruptive behavior as early as possible, ideally during the transition into toddlerhood. Improving our understanding of factors and mechanisms that underlie children’s disruptive behavior can pave the way for more effective preventive intervention.

To understand the development of child disruptive behavior, it is important to consider its key determinants. Parenting practices have been identified as the most important contributor to changes in disruptive behavior (Boeldt et al., 2012; Pinquart, 2017). When parents apply harsh and physical disciplining strategies (e.g., yelling, hitting, and humiliating) and lack parental warmth and sensitivity, the likelihood of disruptive behavior in their children increases (Grasso et al., 2016; Brown et al., 2022). In contrast, experiencing warm, supportive, and responsive parenting behavior (e.g., seeing the child’s needs, being able to comfort the child, and complimenting the child) can prevent or decrease disruptive behavior (Davidov and Grusec, 2006; Waller et al., 2014; van Aar et al., 2017). However, little is known about what factors underlie in the link between parenting and children’s disruptive behavior and, in particular, the role of children’s temperament and psychophysiological indicators of stress reactivity in this relationship.

Parenting, negative emotionality and stress reactivity are often seen as independent contributors to the development of disruptive behavior, but the literature also suggests ways in which these constructs are linked across time. For instance, harsh parenting predicts changes in negative emotionality, and negative emotionality predicts changes in disruptive behavior when parenting is warm and supportive (Bates et al., 2019). Yet, there is a remarkable scarcity of longitudinal studies that combine parent-report and child physiological data to study these relations, especially in early-life family situations. It is important to study such early-life family situations, as early childhood is marked by especially rapid physical, motor, cognitive, and emotional regulatory growth (Ainsfeld, 1984; Jennings, 1991). Toddlers begin to explore their environment and learn that their behavior elicits specific responses (Kochanska et al., 1998). This can be challenging for parents, as many of these behaviors involve toddlers’ willful non-compliance (Scaramella and Leve, 2004).

### Relations between parenting practices, negative emotionality and disruptive behavior

An influential factor in children’s development of disruptive behavior is their ability to learn how to regulate emotional responses and related behaviors (Morris et al., 2007). During the first years of life, negative emotionality is considered a core component of temperament (i.e., individual differences in reactivity and self-regulation) (Rothbart and Bates, 2006) and is typically defined as the tendency to easily get distressed, experience more frustration, anger, sadness, and fear (Putnam et al., 2002). According to the tripartite model parents influence their child’s emotion regulation and behavior in several important ways: through modeling their own emotion regulation strategies (e.g., modeling, social referencing), through emotion-related parenting practices (e.g., emotion coaching), and creating a general emotional climate within the family (e.g., attachment, parenting style) (Morris et al., 2017). Even though negative emotionality was originally considered to be a stable construct, it has now been shown that it develops over time (Shiner and Caspi, 2003; Van Aken et al., 2007) and that this development is influenced by parenting (Klein et al., 2018; Huijzer-Engbrenghof et al., 2023). This suggests a mediational path from harsh and unsupportive parenting to more disruptive behavior via an increase in negative emotionality.

Indeed, the literature provides some tentative evidence for this association. For instance, a review found that more psychological control in parenting was related to more negative emotionality across childhood, which was also related to more adjustment problems (Kiff et al., 2011). Further, in a community sample of 306 preschoolers and their mothers, maternal negativity predicted increases in child frustration, which also predicted adjustment problems (i.e., hyperactivity, internalizing, and externalizing problems) when the children were 5 years old (Klein et al., 2018). Furthermore, a study during the COVID-19 lockdown showed that parental verbal hostility was related to increased child emotional dysregulation, leading to more behavior problems (i.e., hyperactivity and inattention) (Marchetti et al., 2020). Finally, in a large longitudinal study, mothers’ depressive symptoms during infancy predicted more adjustment problems when the child was three years old, and infant negative emotionality at six months old predicted more adjustment problems at three years old. However, no mediation path was analyzed for negative emotionality (Dix and Yan, 2014). Even though these studies show some support for a relation between dysfunctional parenting behavior and disruptive behavior via negative emotionality, none of these studies regards parenting as a whole, with both harsh and unsupportive parenting and warm and supportive parenting. Notably, these two dimensions of parenting practices have been shown to be relatively independent of each other (Stormshak et al., 2000; Deater-Deckard et al., 2006), underscoring the importance of including both.

A separate but related literature on child sensitivity traits presents a different picture of how negative emotionality might affect the relation between parenting and child disruptive behavior. A concept that takes centerstage here is Sensory Processing Sensitivity (SPS), which is conceptualized as a biologically-based temperament trait that is characterized by greater awareness of subtle stimuli, deeper cognitive processing of external stimuli, arousability, and higher emotional reactivity (Aron and Aron, 1997). Because of this trait, children high on SPS tend to be more aware of their environment, which influences how they plan, think, and learn. It is believed that their development is more strongly affected by their (parenting) environment (Slagt et al., 2018). It has been found that children high on SPS in a nurturing environment develop greater cognitive and behavioral functioning (Li et al., 2021). Even though negative emotionality is frequently operationalized as a sensitivity trait, Aron and Aron (1997) themselves argued that there is a “high likelihood that negative affect as a personality variable is often the result of an interaction of something like sensitivity with a negative environment” (Aron et al., 2012, p. 271). Thus, negative emotionality can be viewed as a proxy for emotional reactivity, as a part of general responsivity, interacting with exposure to a negative environment such as with dysfunctional parenting. Negative emotionality as a sensitivity trait has been extensively studied under the umbrella of SPS, but the link from parenting to emotional reactivity received less attention. Furthermore, the bio-regulatory processes that go together with emotional reactivity should also be taken into account, as these processes play an important role in the ability and development of emotion regulation (Beauchaine, 2015).

The psychophysiological component of emotion reactivity refers to the flexibility of the body to up or downregulate emotional arousal (Appelhans and Luecken, 2006). A commonly and non-invasive index used to assess emotional reactivity is heart rate variability (HRV), which is the variation in the duration between subsequent heartbeats and provides information on how the parasympathetic nervous system (PNS) influences heart functioning (Porges, 2001). The PNS is part of the autonomic nervous system (ANS) and is responsible for restoring and protecting our energy levels and vital organs. When the ANS is activated, it will slow the heart rate and increase HRV. The opposing system is the sympathetic nervous system (SNS), which is responsible for adaptive responses to external stimuli. Activation of the SNS will accelerate heart rate and decrease HRV. The ANS and SNS continuously interact with each other and govern the body’s capacity and flexibility to regulate emotions (Appelhans and Luecken, 2006). Typically, HRV increases with age from infancy through middle childhood, indicating that the PNS matures over time (Patriquin et al., 2015). This increase is also in line with the expansion of emotion regulation as children gain in motor, communication and cognitive skills (Sroufe, 1997).

When parents struggle to regulate their own emotions, this can hinder their parenting behavior, resulting in harsh and unsupportive parenting, affecting the child’s emotion regulation and behavior in turn (Morelen et al., 2016). In contrast, when parents are capable of guiding their child through emotional states, the child develops a healthy physiological emotion regulation (Zeegers et al., 2018). Studies have shown that a stressful family environment (i.e., harsh and unsupportive parenting) can dysregulate the PNS and SNS systems, affecting children’s sensitivity to the dysfunctional parenting environment (Oshri et al., 2020). When children’s stress response system is repeatedly triggered, the system will become continuously overactivated, and children will develop a chronically upregulated stress reactivity and lowered self-regulatory capacity, resulting in a low and/or excessive reactivity in HRV (Beauchaine, 2015; Wesarg et al., 2022). It has also been shown that HRV is predictive of disruptive behavior and later psychopathology (Hinnant and El-Sheikh, 2013). For instance, in toddlers, high HRV during rest has been related to social competence, empathy, regulation of distress during frustrating events, lower levels of aggression and greater attention control (Beauchaine, 2015). Furthermore, based on a meta-analysis with children ranging from toddlers to adolescents, lower levels of HRV decrease during a stressful event were linked to more externalizing behavior, whereas greater levels of HRV decrease during a stressful event were associated with fewer externalizing behaviors (Graziano and Derefinko, 2013). To our knowledge, there are no studies in which the role of toddler’s negative emotionality and physiological stress reactivity are simultaneously investigated in the relation between parenting and disruptive behavior.

### The present study

In this study, we investigated whether the longitudinal association between parenting practices (harsh and unsupportive parenting and warm and supportive parenting) and child disruptive behavior in toddlerhood would be mediated by children’s negative emotionality and stress reactivity (HRV). We expected that harsh and unsupportive parenting would lead to more disruptive behavior via increases in children’s negative emotionality and lower levels of HRV stress reactivity over time. In contrast, we expected that warm and supportive parenting would lead to lower levels of disruptive behavior via less negative emotionality and higher levels of HRV stress reactivity over time. Because disruptive behavior is a forerunner of clinical diagnoses of externalizing disorders (Caspi et al., 2020), and toddlers usually do not get diagnosed this young, we chose to adopt the term disruptive behavior (i.e., disobedience, defiance of authority, an angry or irritable mood state, and verbal or physical aggression toward others).

## Method

### Procedure

Participating families took part in a longitudinal study: Joint (Epi)genetics Of Parenting And Stress-Reactivity in the Development of Youth (JEOPARDY; Overbeek et al., 2020). The study was approved by the Ethical Review Board of the Department of Child Development and Education at the University of Amsterdam (2019-CDE-10160). The families were recruited through the Amsterdam SARPHATI cohort — a large dynamic cohort study that systematically monitors children’s health and development from birth up to 18 years of age (Ujcic-Voortman et al., 2020) — while others were drawn from the general population within or around the municipality of Amsterdam by distributing flyers in daycare centers, playgrounds, and parks and by placing adverts on social media. Families with toddlers aged between 12–16 months at the time of recruitment could participate in the study. Parents participating in the SARPHATI cohort were administered the Dutch version of the Parenting Stress Questionnaire–Short (Opvoedingsbelastingsvragenlijst-kort, OBVL-k; Vermulst et al., 2012). All parents who scored above the 75^th^ percentile on this questionnaire, indicating heightened levels of parenting stress, were invited to participate in JEOPARDY. Families recruited via flyers were screened for parenting stress through the same questionnaire, but could participate regardless of their score. Overall, this led to a sample of ‘at-risk families’ with high parenting stress (*n* = 56), and therefore at higher risk of harsh parenting, and ‘low-risk families’ without elevated levels of parenting stress (*n* = 16). All families received an information letter and a phone call explaining the study in more detail. Parents had to be at least 18 years old and master the Dutch language, and it was required that both child and parent had no physical or mental health condition that would impede taking part in the test sessions. Of the 108 families that showed interest, 72 (67%) decided to participate in the study.

After inclusion, families were visited in their homes for assessments every six months across five measurement waves. This study reports on the first three of those waves, as the study focusses on early family dynamics. Because some families (*n* = 10 intervention, *n* = 12 active control condition) in our sample participated in a parenting intervention, they followed a different timeline, with follow-up assessments after the baseline at 3 –months and 6 –months (instead of 6 months and 6 months)—we controlled for these differing time intervals alongside intervention status in the analyses (see strategy for analyses).

At each measurement wave, the families were visited in their homes for an assessment (except for 32 families during the first wave due to COVID-19 restrictions, for whom assessments were conducted online via Zoom). During the first assessment, the trained experimenter and assistant took the time to explain the study in more detail, answered questions, and collected the signed informed consent letter. Next, a series of tasks to assess stress reactivity, alongside other tasks not relevant to the present study, were executed. Furthermore, parents filled in a set of online questionnaires, which were emailed to them prior to each home visit. This procedure was repeated at each measurement wave. Parents received a gift certificate during each home visit with a value of up to €50 in total.

### Participants

In total, 72 parent-toddler dyads participated in the study, which comprised a highly intense data collection procedure. Toddlers were between 12 and 16 months old at T1 (*M_age_* = 14.68, SD = 2.12); 51.4% were girls and mostly Dutch (84.7%). Participating parents (88.9% mothers) were aged between 25 and 44 years old (*M_age_* = 35.54, SD = 3.50) and were also mostly Dutch (73.6%). Twenty-one percent were from other countries, such as United States, Germany and Turkey. The parents were mostly (55.6%) married or had a registered partnership (36.1% had a relationship, and 5.6% were single). Furthermore, 66.7% of the parents had completed a university-level educational degree, 29.2% a higher vocational educational degree, and 2.8% completed high school or middle vocational educational degree. Nearly all parents were employed (91.7%) and had a family income of over €3.200 net(t) per month.

### Measures

#### Toddler disruptive behavior

To assess disruptive behavior, the Dutch version of the Child Behavior Check List (CBCL; Achenbach and Rescorla, 2000) for young preschoolers (1,5–5 years) was used. The questionnaire can adequately measure children’s problem behavior from an early age (Koot et al., 1997). Parents rated their child’s behavior from the past two months on a three-point scale (“1 = not true, 2 = sometimes / a little, and 3 = very often/true) on 99 questions on emotional and behavioral problems. For this study, we used the sum score of the externalizing broadband score, which is comprised of the syndrome scales Attention problems (5 items) (i.e., “Cannot sit still, is restless or hyperactive”) and Aggressive behavior (19 items) (i.e., “Is easily upset when things do not go his/her way”). The externalizing scale achieved good reliability. Cronbach’s alpha’s were α = 0.91 at T1, α = 0.90 at T2, and α = 0.89 at T3, respectively.

#### Parenting

Parenting practices were assessed with the Dutch version of the Comprehensive Early Childhood Parenting Questionnaire (CECPAQ; Verhoeven et al., 2017) and were used in the analyses at the first and second wave. The questionnaire is a good measure of self-perceived parenting behavior for parents with young children (Verhoeven et al., 2017). Parents were asked to indicate on a Likert scale ranging from 1 to 6 (1 = *never*, 6 = *always*) whether the statement was applicable to them/their child. For warm and supportive parenting, the subscales Support (13 items) (i.e., “I tell my child how happy s/he makes me”) and Positive Discipline (4 items) were used (i.e., “I explain to my child why certain rules must be followed”). For harsh and unsupportive parenting, the subscale Harsh Discipline (12 items) was used (e.g., “When my child disobeys, I get angry and raise my voice”). Item scores were averaged to obtain total subscale scores, with higher scores representing higher levels of either warm/supportive parenting or harsh/unsupportive parenting practices. In line with former findings (Verhoeven et al., 2017), warm and supportive parenting achieved good reliability at T1 (*α* = 0.88), T2 (*α* = 0.87), and T3 (*α* = 0.86). Due to poor reliability within the Harsh Discipline subscale we dropped one item (i.e., “When my child misbehaves… I raise my voice or yell / I speak to my child calmly”). For the final scale, Cronbach’s alphas were adequate (*α* = 0.73 at T1, *α* = 0.80 at T2, and *α* = 0.79 at T3).

#### Negative emotionality

Negative emotionality was assessed with the Dutch version of the 12-item negative emotionality scale of the Early Childhood Behavior Questionnaire Very Short Form (ECBQ-VSF; Putnam and Rothbart, 2006), and was used in the analyses at the second and third wave. The questionnaire is a valid measure to assess child temperament between the ages of 18 and 36 months (Putnam and Rothbart, 2006). Parents were asked to indicate the frequency with which they observed a certain behavior over the past two weeks based on a Likert scale ranging from 1 to 7 (e.g., “When s/he was upset, how often did your child cry for more than 3 min, even when being comforted?”). Item scores were averaged to obtain a total score, with higher scores representing higher levels of negative emotionality. In line with expectations, Cronbach’s alpha’s negative emotionality scale increased as the children’s age increased (*α* = 0.48 at T1, *α* = 0.69 at T2, and *α* = 0.69 at T3).

#### HRV stress reactivity

The Laboratory Temperament Assessment Battery (Lab-TAB; Goldsmith and Rothbart, 1999),were conducted during home visits to assess stress reactivity. Parents were asked to place a Polar H7 heart rate sensors, which had been adapted with Velcro for use within a pediatric population, around their child’s chest. The Polar device was connected via Bluetooth to the laptop containing Vsrrp98 software, used to register HRV data during the tasks (Molenkamp, 2011). A second laptop containing Presentation software was connected to the first laptop with a glass fiber amplifier. The second laptop registered the triggers, cued by the lead experimenter, of each task’s start and finish point within the experiment. The task started with the child seated in a high chair with the parent sitting right behind the child. For the first 3 min, the child watched a Miffy video clip on a tablet, during which baseline measure was registered. Next, the assistant entered the room wearing an animal mask and approached the child at intervals (total time 1:10 min). In two subsequent episodes, the assistant re-entered the room with a remote-controlled spider that performed a series of movements (total time approx. 1:15 min), and a remote-controlled robot was placed on the floor in front of the child that also performed a series of movements (total time 1:45 min). Finally, the tablet with the Miffy video clip was replaced in front of the child for a recovery phase that lasted 3 min. If the child cried or became upset during the tasks for longer than 30 s, the parent was allowed to comfort the child and decide whether the experiment would be continued or aborted.

Data from the Polar heart rate monitor was received by the measurement software as interbeat intervals. The Polar sensor sends data every second, and when no movement artefacts are detected. Therefore, the data was realigned before data processing by the software (Vrssp98, version 12.9, 2024). After realigning the data, the program calculated heart rate and heart rate variability from the individual interbeat interval, using a criterion of IBI < last IBI + 33% and IBI > last IBI – 33%. HRV was calculated as the Root Mean Square of Successive Differences in IBI (RMSSD). After processing, the HR and HRV data were manually checked for noisy data (i.e., movement and/or crying of the child and technical issues) in which values did not match plausible HR and HRV for this age group. Based on previous research, a cut-off of an HR below 95 (De Nederlandse Hartstichting, n.d.) and an HRV above 100 (Zeegers et al., 2018; Nikolic et al., 2022) was made. Within each phase, when these values were present, they were deleted. A minimum of 30 consecutive seconds of measurement units had to be present in the specific phase to score. For more details, see Appendix A. For baseline HRV, the mean value of the HRV during the three-minute baseline was used. To assess HRV stress reactivity, the mean value of the baseline HRV was subtracted from the mean value of the three stress tasks combined (i.e., mask, spider, robot). Negative values indicate a decrease in HRV during the stress tasks. For our analyses, we used data from the second and third wave.

### Analyses

Given the ubiquity of missing data due to attrition in longitudinal designs, as expected, there were more missing data at T3 (*n* = 18, 18 and 17 missing values, 12.9, 12.9, and 12.2%) and T2 (*n* = 11, 10, and 9 missing values, 7.9, 7.2, and 6.5%) than there were at T1 (*n* = 1, 1 and 1 missing values, 0.7%) for the parenting, disruptive behavior, and negative emotionality items. For the HRV stress reactivity, there were 40 missing values at T1 (28.8%), 37 at T2 (26.64%) and 30 at T3 (21.6%). Four families actively resigned from participating in the study (due to moving to a different region or a new pregnancy, for instance), and 14 families dropped out after contacting them several times without success. Before describing our target analyses, we explain how incomplete data were handled.

Multiple imputation is a method that preserves all available information observed in our sample, preventing unnecessary loss of power by replacing missing values with a distribution of plausible values (Little et al., 2014). Multiple imputation assumes data are missing at random (MAR) or missing completely at random (MCAR). A Little’s MCAR test confirmed this to be the case for the current study data (*χ*^2^ = 2826.612, df = 20,990, *p* = 1.00). We used SPSS (version 29) to impute missing values for all three waves of the data ten imputed datasets were constructed. The data were imputed with predictive mean matching so that the only values chosen to replace missing values were among the observed values within individuals. The auxiliary variables were all items from the measures used for analyses. After imputing the missing item-level data, scale scores were computed (i.e., passive imputation of scale scores).

To answer the research questions, we ran partial mediation analyses using Hayes’ PROCESS macro (Hayes, 2017), model 4 in SPSS (version 29) to examine whether negative emotionality and/or HRV stress reactivity mediated the association between parenting and disruptive behavior. The variables were standardized into Z-scores and we used bootstrapping with 5.000 samples redrawn to estimate the 95% confidence intervals for the mediation effect. Furthermore, the results were pooled across multiple imputations in Excel using Rubin’s rules (Rubin, 1987). PROCESS uses a regression-based approach and allows for comparison of indirect effects, effect size, and examines the total effect model. PROCESS estimates the indirect coefficient for each indirect pathway between the independent variable (harsh parenting or warm and supportive parenting) and the dependent variable (disruptive behavior), accounting for respective indirect effects (negative emotionality or HRV stress reactivity). We tested eight models in total as we examined the longitudinal model for both harsh parenting and warm and supportive parenting as predictors at T1. As toddlers go through rapid developmental changes, parenting also undergoes a transformation (Scaramella and Leve, 2004). To capture this transformation, we examined the longitudinal model with parenting as a predictor at T2 as well. In the T1 parenting models, the mediator variables for negative emotional reactivity and HRV stress reactivity were taken from the T2 assessment. In the T2 parenting models, the mediator variables for negative emotional reactivity and HRV stress reactivity were taken from the T3 assessment, allowing for a longitudinal analysis of the linkages with parenting measured one wave earlier. As children at this young age develop at different paces in which a 4 months age difference can be impactful (Sheldrick et al., 2019) we controlled for age. We also controlled for intervention status and baseline disruptive behavior, because of the differences in timeline and some families receiving an intervention.

## Results

**Figure 1:**
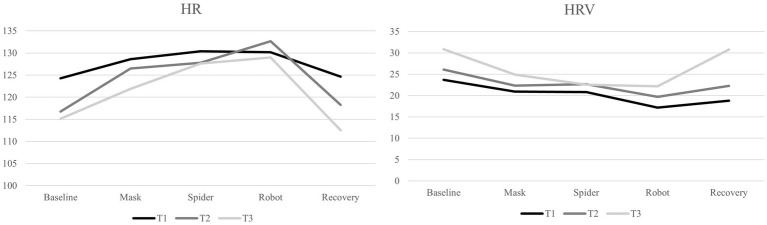
HR and HRV across Lab-TAB phases.

Table 1 shows the means, standard deviations and min-max values of the variables at all three timepoints. A repeated Measure ANOVA showed that harsh parenting significantly increased over time (see Table 1). Also, toddlers showed significant increases of disruptive behavior and negative emotionality over time. HRV significantly decreased from T1 to T3 (see Figure 1). A manipulation check with paired-sample *T*-tests revealed that the stress induction was successful. For wave 2, there was a significant difference between the HRV stress phases (*M* = 21.48, *SD* = 6.79) and the HRV baseline (*M* = 26.06, *SD* = 9.00); *t* (988^1^) = −5.47, *p* < 0.001, *d* = 7.06 with a 95% CI ranging from −6.22 to −2.94. The same was true for wave 3, with a significant difference between the HRV stress phases (*M* = 23.19, *SD* = 6.79) and the HRV baseline (*M* = 30.70, *SD* = 9.26); *t* (988^1^) = −6.95, *p* < 0.001, *d* = 8.55 with a 95% CI ranging from −9.62 to −5.38. Furthermore, we checked whether there was a significant difference in our sample between the risk and non-risk families concerning parenting practices, which was indeed the case harsh parenting at T1 [*F* = 6.01, *t* (1624^1^) = −2.96, *p* = 0.003], warm/supportive parenting at T1 [*F* = 0.10, *t* (453^1^) =3.27, *p* = 0.001]. Risk families scored higher on harsh parenting compared to the non-risk families, which scored higher on warm/supportive parenting. Overall, the distribution of scores on both parenting practice measure was adequate.

**Table 1:** Descriptive statistics.

		Mean	SD	Min-Max	*F* Min-Max	Eta Squared
Harsh parenting	T1	1.71	0.69	1.33;2.58		
	T2	1.78	0.71	1.42;3.00		
	T3	1.85	0.64	1.42;3.00	6.34–10.39*	0.8–0.13
Warm/sup. parenting	T1	4.83	0.28	2.79;5.85		
	T2	4.85	0.29	3.46;5.88		
	T3	4.88	0.31	3.89;5.72	0.09–0.9	0.001–0.01
Disruptive behavior	T1	37.77	0.59	25;60		
	T2	39.34	0.53	24;59		
	T3	41.05	0.43	25;56	6.20–8.34*	0.08–0.10
Neg. Emotionality	T1	2.36	8.21	1.09;4.91		
	T2	2.80	7.42	1.36;5.27		
	T3	2.88	6.75	1.64;4.82	26.64–30.51*	0.27–0.30
HRV-SR	T1	4.03	5.84	−14.16;20.30		
	T2	4.58	6.94	−14.32;27.36		
	T3	7.50	8.70	−10.85;36.49	3.76–7.36*	0.05–0.09

Pooled across imputed datasets. *p < 0.05. Warm/Sup. Parenting = Warm and Supportive Parenting. Neg. Emotionality = Negative emotionality. HRV-SR = heart rate variability stress reactivity.

Correlations between all variables are presented in Table 2. Notably, harsh parenting T1 did not correlate significantly with either disruptive behavior, negative emotionality, or HRV stress reactivity across all time points. For warm and supportive parenting T1 there was a significant negative association with disruptive behavior T3. Harsh parenting T2 showed a significant correlation with disruptive behavior T2 and T3, along with negative emotionality T3. Warm and supportive parenting T2 showed a significant negative association with negative emotionality T3. Furthermore, negative emotionality T2 significantly correlated with disruptive behavior T2 and T3. The same was true for negative emotionality T3 and disruptive behavior T3. Finally, HRV stress reactivity T2 showed a significant association with negative emotionality T2. All significant correlations were weak to moderate and in the expected direction.

**Table 2:** Correlations between parenting, disruptive behavior, negative emotionality and HRV stress reactivity over time.

	1.	2.	3.	4.	5.	6.	7.	8.	9.	10.	11.	12.	13.	14.	15.
1. Harsh Par. T1	-														
2. Harsh Par. T2	0.447**	-													
3. Harsh Par. T3	0.490**	0.579**	-												
4. Warm Par. T1	−0.326**	−0.252*	−0.258*	-											
5. Warm Par. T2	−0.283*	−0.234	−0.144	0.639**	-										
6. Warm Par. T3	−0.286*	−0.150	−0.241*	0.553**	0.651**	-									
7. Dis behavior T1	0.212	0.192	0.166	−0.225	−0.124	−0.025	-								
8. Dis behavior T2	0.021	0.315**	0.122	−0.150	−0.263*	−0.076	0.647**	-							
9. Dis behavior T3	0.105	0.319*	0.397**	−0.258*	−0.176	−0.208	0.468**	0.513**	-						
10. Neg. Emo T1	0.170	0.110	0.169	0.008	0.117	−0.040	0.436**	0.141	0.118	-					
11. Neg. Emo T2	0.150	0.201	0.28*	0.019	0.023	−0.073	0.324**	0.240*	0.206	0.624**	-				
12. Neg. Emo T3	0.176	0.286*	0.301*	−0.150	−0.061	−0.262*	0.302*	0.263*	0.399**	0.397**	0.637**	-			
13. HRV-SR T1	−0.04	−0.129	−0.065	−0.012	0.090	0.045	0.026	−0.056	−0.016	−0.023	−0.117	−0.151	-		
14. HRV-SR T2	−0.059	−0.066	−0.064	0.051	−0.067	−0.035	0.090	0.023	0.087	0.102	0.266*	0.172	−0.063	-	
15. HRV-SR T3	−0.22	−0.33	0.080	−0.62	0.019	0.188	−0.008	−0.074	0.055	−0.081	−0.031	−0.177	0.143	0.076	-

Pooled across imputed datasets. Stars indicate significance level (* < 0.05, ** < 0.01). Harsh Par. = harsh parenting. Warm Par. = warm parenting. Dis. Behavior = disruptive behavior. Neg. Emo. = Negative emotionality. HRV-SR = heart rate variability stress reactivity.

### Mediation of negative emotionality

We tested the mediation of negative emotionality T2 in the relation between harsh parenting T1 and disruptive behavior T3. Harsh parenting at T1 did not significantly predict disruptive behavior at T3 in the total and direct models. Moreover, there were no significant indirect effects, indicating that negative emotionality T2 did not mediate an effect of harsh parenting T1 on disruptive behavior T3. The same results emerged when we tested the model with warm and supportive parenting T1 as predictor (see Table 3).

**Table 3:** Mediation results of parenting T1 on negative emotionality T2, and disruptive behavior T3.

	*β*	SE	*t*	*p*	CI
**Harsh parenting**					
Harsh parenting ➔ negative emotionality	0.13	0.12	1.09	0.306	−0.15;0.41
Negative emotionality ➔ disruptive behavior	0.08	0.12	0.64	0.539	−0.20;0.36
Direct: harsh parenting ➔ disruptive behavior	−0.01	0.12	−0.05	0.958	−0.28;0.26
Total: harsh parenting ➔ disruptive behavior	0.004	0.12	0.03	0.973	0.26;0.27
Indirect effect: harsh parenting ➔ negative emotionality ➔ disruptive behavior	0.01	0.03	0.37	0.722	−0.05;0.08
**Warm and supportive parenting**					
Warm/supportive parenting ➔ negative emotionality	0.06	0.12	0.53	0.611	−0.21;0.34
Negative emotionality ➔ disruptive behavior	0.09	0.12	0.73	0.483	−0.18;0.36
Direct: warm/supportive parenting ➔ disruptive behavior	−0.16	0.11	−1.39	0.199	−0.42;0.10
Total: warm/supportive parenting ➔ disruptive behavior	−0.15	0.11	−1.34	0.213	−0.41;0.11
Indirect effect: warm/supportive parenting ➔ neg. Emo. ➔ disruptive behavior	0.02	0.04	0.40	0.695	−0.08;0.11

Pooled across imputed datasets. Age, Intervention status, and disruptive behavior T1 were used as control variables.

Next, we tested the mediation of negative emotionality T3 on the relation between harsh parenting T2 and disruptive behavior T3. Harsh parenting T2 did not significantly predict disruptive behavior at T3yet, the association between negative emotionality T3 and disruptive behavior was significant T3 (*β* = 0.26, *SE* = 0.11, *t* = 2.36, *p* = 0.043). However, when Bonferroni correction was applied to adjust for multiple testing, setting the significance threshold at *α* = 0.006, the association was no longer significant. Within the relation between harsh parenting T2 and disruptive behavior T3 with negative emotionality T3 as mediator, results showed no mediation. The model was repeated with warm and supportive parenting as predictor. The direct association between warm and supportive parenting T2 and disruptive behavior T3 was not significant. However, the direct association between negative emotionality T3 and disruptive behavior T3 was significant (*β* = 0.31, *SE* = 0.11, *t* = 2.82, *p* = 0.020). Here too the Bonferroni correction led to the disappearance of the significant association. The longitudinal association between warm and supportive parenting T2 and disruptive behavior T3 with negative emotionality T3 as mediator showed no mediation (see Table 4).

**Table 4:** Mediation results of parenting T2 on negative emotionality T3, and Disruptive Behavior T3.

	*β*	SE	*t*	*p*	CI
					
**Harsh parenting**					
Harsh parenting ➔ negative emotionality	0.24	0.12	1.96	0.082	−0.04;0.51
Negative emotionality ➔ disruptive behavior	0.26	0.11	2.36	0.043	0.01;0.52
Direct: harsh parenting ➔ disruptive behavior	0.20	0.12	1.70	0.124	−0.07;0.46
Total: harsh parenting ➔ disruptive behavior	0.26	0.12	2.24	0.052	−0.002;0.52
Indirect effect: harsh parenting ➔ negative emotionality ➔ disruptive behavior	0.06	0.04	1.42	0.190	−0.04;0.16
**Warm and supportive parenting**					
Warm/supportive parenting ➔ negative emotionality	−0.03	0.12	−0.24	0.817	−0.30;0.24
Negative emotionality ➔ disruptive behavior	0.31	0.11	2.82	0.020	0.06;0.56
Direct: warm/supportive parenting ➔ disruptive behavior	−0.12	0.11	−1.07	0.314	−0.36;0.13
Total: warm/supportive parenting ➔ disruptive behavior	−0.13	0.11	−1.11	0.297	−0.38;0.13
Indirect effect: warm/supportive parenting ➔ neg. Emo. ➔ disruptive behavior	−0.01	0.04	−0.20	0.845	−0.11;0.09

Pooled across imputed datasets. Age, Intervention status, and Disruptive Behavior T1 were used as control variables. Neg. Emo. = negative emotionality.

### Mediation of HRV stress reactivity

Concerning our hypothesis with HRV stress reactivity as a mediator, we tested whether parenting predicted disruptive behavior via HRV stress reactivity. Results showed that harsh parenting T1 did not significantly predict disruptive behavior at T3. Moreover, there were no significant indirect effects, indicating that HRV stress reactivity did not mediate an effect of harsh parenting T1 on disruptive behavior T3. The same was true when we repeated the model with warm and supportive parenting T2 as predictor (see Table 5).

**Table 5:** Mediation results of Parenting T1 on HRV T2, and disruptive behavior T3.

	*β*	SE	*t*	*p*	CI
**Harsh Parenting**					
Harsh parenting ➔ HRV	−0.11	0.14	−0.80	0.446	−0.41;0.20
HRV ➔ disruptive behavior	0.04	0.12	0.34	0.743	−0.23;0.31
Direct: harsh parenting ➔ disruptive behavior	0.01	0.12	0.07	0.948	−0.26;0.28
Total: harsh parenting ➔ disruptive behavior	0.004	0.12	0.03	0.973	−0.26;0.27
Indirect effect: harsh parenting ➔ HRV ➔ disruptive behavior	−0.004	0.02	−0.22	0.829	−0.04;0.04
**Warm and supportive parenting**					
Warm/supportive parenting ➔ HRV	0.09	0.13	0.69	0.505	−0.21;0.39
HRV ➔ disruptive behavior	0.05	0.12	0.45	0.664	−0.21;0.31
Direct: warm/supportive parenting ➔ disruptive behavior	−0.16	0.11	−1.37	0.204	−0.42;0.10
Total: warm/supportive parenting ➔ disruptive behavior	−0.15	0.11	−1.34	0.213	−0.41;0.10
indirect effect: warm/supportive parenting ➔ HRV ➔ disruptive behavior	0.005	0.02	0.22	0.829	−0.04;0.05

Pooled across imputed datasets. Age, intervention status, and disruptive behavior T1 were used as control variables. HRV = heart rate variability.

Finally, we tested whether parenting at T2 predicted disruptive behavior at T3 via HRV stress reactivity at T2. Results showed that harsh parenting T2 did not significantly predict disruptive behavior at T3. There were also no significant indirect effects, indicating that HRV stress reactivity at T2 did not mediate an effect of harsh parenting at T2 on disruptive behavior T3. Likewise, there were no significant direct and indirect effects when warm and supportive parenting at T2 was used in the mediation analyses (see Table 6).

**Table 6:** Mediation results of parenting T2 on HRV T3, and disruptive behavior T3.

	*β*	SE	*t*	*p*	CI
**Harsh parenting**					
Harsh parenting ➔ HRV	−0.01	0.13	−0.07	0.946	−0.29;0.28
HRV ➔ disruptive behavior	0.03	0.11	0.30	0.771	−0.22;0.29
Direct: harsh parenting ➔ disruptive behavior	0.26	0.12	2.23	0.053	−0.004;0.52
Total: harsh parenting ➔ disruptive behavior	0.26	0.11	2.24	0.052	−0.002;0.52
Indirect effect: harsh parenting ➔ HRV ➔ disruptive behavior	0.00	0.02	0.00	0.997	−0.05;0.05
**Warm and supportive parenting**					
Warm/supportive parenting ➔ HRV	0.02	0.13	0.14	0.895	−0.27;0.30
HRV ➔ disruptive behavior	0.03	0.12	0.30	0.774	−0.23;0.30
Direct: warm/supportive parenting ➔ disruptive behavior	−0.13	0.11	−1.11	0.297	−0.38;0.13
Total: warm/supportive parenting ➔disruptive behavior	−0.13	0.11	−1.11	0.297	−0.38;0.13
Indirect effect: warm/supportive parenting ➔ HRV ➔ disruptive behavior	0.001	0.02	0.05	0.958	−0.05;0.05

Pooled across imputed datasets. Age, intervention status, and disruptive behavior T1 were used as control variables. HRV = heart rate variability.

## Discussion

Harsh and unsupportive parenting is a risk factor for the development of disruptive behavior in children. However, little is known about how children’s temperament and stress reactivity influence this relation. More specifically, there are no studies in which the role of toddler’s negative emotionality and physiological stress reactivity are simultaneously investigated in the relation between parenting and disruptive behavior. In this three-wave longitudinal study, we investigated the longitudinal association between parenting practices (harsh and unsupportive parenting and warm and supportive parenting) and child disruptive behavior and whether this association is mediated by children’s negative emotionality and emotional stress reactivity (HRV). Overall, we found that negative emotionality and stress reactivity did not mediate the relation between parenting and disruptive behavior.

Contrary to our hypothesis, in this sample of toddlers and their parents we found no proof that harsh parenting predicts disruptive behavior, neither directly nor indirectly via negative emotionality. Even though we expected to find this relation, specifically with a measure of negative emotionality as a proxy for a general responsivity tendency in infants—in accordance with Sensory Processing Sensitivity conceptualizations (Aron et al., 2012)—we did not find this link. A possible explanation might be that the parenting behavior in the families studied was, in most cases, “good enough parenting” (Winnicott, 1960)—which is to say that generally levels of parental harshness in this sample were quite low. Winnicott, in his days, stated that as long as parents are reliable and the child is well-cared for, parents’ minor ‘failures’ ultimately foster independence and autonomy in the growing child. When considering the average score for harsh parenting in our sample, they were quite low, whereas the average score for warm and supportive parenting was relatively high. This might indicate that the parenting behavior was indeed good enough and did not cause disruptive behavior at this early age.

Another explanation is that we measured relatively low levels of harsh parenting and high levels of warm/supportive parenting due to the characteristics of our sample. Although we included mainly higher-risk families by screening them on parenting stress, the risk criterion was set relatively low (i.e., 75th percentile on the OBVL) so that many different parents were included—not only those who were highly stressed, but also those who evinced normal and resilient functioning. In addition, the sample consisted mainly of well-educated and relatively high SES families. Compared to high SES parents, parents of low SES are more likely to have additional problems and are, because of that, more at risk for harsh parenting practices (Conger et al., 2010). Perhaps a sample with more variation in SES would also show more variation in parenting practices.

Relatedly, the effects of harsh parenting on child behavior might emerge later in development, as suggested by research on coercive cycling in families (Patterson, 2002). A longitudinal study among 731 parent–child dyads that examined coercive interactions at ages 2, 3, 4, and 5 showed that coercive interactions in early childhood predicted disruptive behaviors in the years to come (Smith et al., 2014), which also seems to continue into adolescence, as externalizing problems increase when parents exert psychological control on their child (Pace et al., 2018).In our sample, both harsh parenting and disruptive behavior increased significantly over time. A meta-analysis on parenting dimensions and their effect on externalizing problems in children and adolescents found that the association between parenting and externalizing problems were stronger in older samples (Pinquart, 2017). It is thus possible that these associations are not detectable in our sample of young children yet. Future research with more measurement waves can shed light on the development of disruptive behavior from toddlerhood into the (pre)school period and adolescence in relation to parenting practices.

Even though warm and supportive parenting scores were high on average, this did not predict fewer disruptive behavior. Moreover, negative emotionality and HRV stress reactivity did not mediate in this relationship. However, correlations showed that warm and supportive parenting at wave 1 was associated with less disruptive behavior at wave 3. Also, warm and supportive parenting at wave 3 was associated with less negative emotionality at wave 3. Even though we expected to find that warm and supportive parenting would predict less disruptive behavior over time (Davidov and Grusec, 2006; Waller et al., 2014), we did not find the same results. Perhaps the toddler’s young age contributes to the fact that we found no predictive results yet. When considering the developmental cascade model, several methodological issues may apply to our study. For instance, a longer-term analysis may be required to detect a cascade effect. While our design is indeed longitudinal, it might take a longer period than the18 month interval assessed in this study to see how parenting affects child behavior, specifically at this young age. Furthermore, the timing of the assessments is also of importance. Correlations between variables might obscure a cascade effect if they are too close together (Masten and Cicchetti, 2010). As with the null finding on harsh parenting, in which we suggest extending the measurement waves over a longer period, the same might be true to detect an effect of warm and supportive parenting on disruptive behavior.

As for the hypothesis on stress reactivity, we found no proof that harsh parenting predicts disruptive behavior, neither directly nor indirectly via HRV stress reactivity. Even though a meta-analysis on HRV withdrawal during challenging states and children’s adaptive functioning found that lower levels of HRV withdrawal were linked to more externalizing behavior problems (Graziano and Derefinko, 2013), we did not find the same results. A possible explanation might be that the children in the meta-analysis were mainly of school age. Their PNS will have matured further than the toddlers in our sample, which will also influence their ability to regulate emotions.

While we investigated the relation from parenting to disruptive behavior (via negative emotionality and/or HRV stress reactivity), it is important to acknowledge that parenting does not happen in isolation. Even though there is numerous evidence that shows how parenting practices affect the development of disruptive behavior (Hoeve et al., 2009; Pinquart, 2017; van der Storm et al., 2022), there is also support for a bidirectional relation. Many scholars have, time and again posed that child disruptive behavior both elicits harsh parenting and suppresses the use of warm and supportive parenting, which subsequently escalates the disruptive behavior even further (Patterson, 1982, 2002). Many studies on these coercive patterns consist of school-aged children and adolescents. However, the coercive cycle might look different for younger children compared to older children, as young children rely more strongly on their parents for emotion regulation for instance (Pardini et al., 2008). Future research, similar to our study, yet with a larger sample and adequate statistical power, and over a longer period of time, would be very much suited for studying the bidirectional associations between parenting and child disruptive behavior and negative emotionality in toddlerhood.

### Strengths and limitations

The current study has several strengths. First, we examined the relation between parenting and disruptive behavior using longitudinal data, which gives insight into changes over time, as well as into developmental trajectories. Furthermore, the study made use of a multi-source measurement approach, with parents self-reports being combined with physiological data from children. This enabled us to examine whether child behavior is related to physiological responses (i.e., HRV) and whether parenting affects these responses in toddlers. Moreover, the patterns of HRV during stress inductions and how these patterns develop as toddlers get older are very valuable to the field in itself. We showed that the performed stress induction was successful in this young age group and that the Polar belt is a sound and noninvasive technique to obtain HRV data.

One of the primary limitations of this study is the small sample size and lower statistical power. This limits the generalizability of the findings and increases the likelihood of Type II errors, meaning some true effects may not have been detected. Thus, caution is needed when interpreting the results. Because of the small sample, the options for using more sophisticated SEM analytical approaches were also limited. Ideally, in future studies the longitudinal relation between parenting and disruptive behavior and the role negative emotionality and stress reactivity play in this relation may be studied with a cross-lagged design. This would enhance the understanding of the directionality and temporal ordering of relationships between these variables, providing stronger evidence of potential causal mechanisms.

Related to the small sample, another limitation was the uneven distribution of at-risk families (i.e., heightened parenting stress, putting the families at risk for harsh parenting) and non-risk families, with the non-risk families being underrepresented. Even though our sample tested significant differences between the two groups in both harsh and warm parenting at wave 1, indicating that there was no restriction of range, the uneven groups (*n =* 56 at-risk versus *n* = 16 non-risk) might have affected the results. Even more so, as all families reported little harsh parenting and relatively high warm parenting. Future research with a more even distribution would make it possible to compare at-risk and non-risk families to strengthen the findings.

Another limitation was the reliability of the scales to measure disruptive behavior and negative emotionality. Because of the young age of the toddlers, especially at the first wave, the reliability was questionable. As time went by, the reliability of the scales improved, which is in line with the validation studies on these questionnaires, as both the ECBQ and the CBCL are deemed reliable from the age of 18 months (Koot,1993; Putnam and Rothbart, 2006). Relatedly, the study only made use of parent-reported measures to assess parenting behavior, disruptive behavior and negative emotionality. Without direct observational data, it is not possible to validate the accuracy of the parent-reported measures. Furthermore, with observational data, measures of disruptive behavior and negative emotionality at this young age could have been more reliably assessed. However, there are also studies that found low agreement between observational and parent-reported measures of child disruptive behavior, indicating a high discrepancy between the two measurement types (Hendriks et al., 2018; Moens et al., 2018). Nevertheless, future research should incorporate both parent-reported and observational methods, especially when studying young children, as measuring child temperament and disruptive behavior via questionnaires alone might not be reliable enough to capture the true essence of the specific behavior.

## Conclusion

The current study aimed to improve our knowledge of what factors underlie in the link between parenting and children’s disruptive behavior and, in particular, the role of children’s temperament and psychophysiological levels of negative emotionality and reactivity in this relationship. What parents reported about their own parenting practices did not predict disruptive behavior of toddlers. Moreover, neither the toddler’s negative emotionality nor their HRV stress reactivity mediated in this relation. However, harsh parenting, negative emotionality and disruptive behavior increased over time, which might indicate that the effects of harsh parenting on child behavior emerge later in development.

## Data availability statement

The raw data supporting the conclusions of this article will be made available by the authors per request.

## Ethics statement

The studies involving humans were approved by the Ethical Review Board of the Department of Child Development and Education at the University of Amsterdam. The studies were conducted in accordance with the local legislation and institutional requirements. Written informed consent for participation in this study was provided by the participants’ legal guardians/next of kin.

## Author contributions

MH-E: Visualization, Conceptualization, Writing – original draft, Project administration, Methodology, Investigation, Formal analysis, Data curation. LR: Writing – original draft, Conceptualization, Supervision. HS: Writing – review & editing, Investigation, Data curation. CW-M: Writing – review & editing, Investigation, Data curation. NC: Writing – review & editing, Investigation. EL: Writing – review & editing, Project administration, Data curation. GO: Methodology, Writing – review & editing, Supervision, Funding acquisition, Conceptualization.

## Footnotes

^1^The *df* for pooled test statistics are a function of *N*, the number of imputations, and the fraction of missing information.
