# Supplementary material for: A longitudinal study on the relation between parenting and Toddler’s disruptive behavior: what is the role of Toddler’s negative emotionality and physiological stress reactivity?
Source: Front Psychol. 2024 Sep 9;15:1444447. doi: 10.3389/fpsyg.2024.1444447 (PMC11418277; doi:10.3389/fpsyg.2024.1444447)
Supplement: Supplementary file 1 [file Data_Sheet_1.docx]

**Appendix**

As described in the Methods, after processing, the HR and HRV data were manually checked for noisy data. Noisy data are data in which values did not match plausible HR and HRV for this age group. Based on previous research, a cut-off of an HR below 95 (Nederlandse Hartstichting, n.d.) and an HRV above 100 (Nikolic et al., 2022; Zeegers et al., 2018) was made. This noisiness can be caused by movement and/or crying of the child or technical issues, such as a failing Bluetooth connection between the Polar device and the laptop.
 The noisy data was checked per data point. In the processed data file, each data point is the average of HR or HRV for every 10 seconds. The baseline and recovery phases consisted of 18 data points each (the duration of the tasks was 3 minutes = 180 seconds, 180 seconds / 10 = 18 datapoints). The mask, spider and robot phases each consisted of zero to 16 data points, depending on the duration of each phase. The duration of these phases varied as some toddlers cried during specific tasks, and the task was aborted, or the phase was skipped altogether if the parent decided to abort the experiment. When invalid values were present within a phase, they were flagged in the data file. If an invalid HR value (i.e., below 95) was flagged, the corresponding HRV value was also flagged. This resulted in the following number of cases per phase in which one or more values were flagged as invalid (Table A1).

**Table A1** *Number of cases with one or more invalid values per phase*

|  | Total N | Baseline | Mask | Spider | Robot | Recovery |
| --- | --- | --- | --- | --- | --- | --- |
| HRV T1 | 37 | 7 | 7 | 4 | 5 | 9 |
| HRV T2 | 41 | 10 | 8 | 7 | 4 | 11 |
| HRV T3 | 45 | 12 | 6 | 8 | 8 | 15 |
| *Note. HRV = heart rate variability* | | | | | | |

Next, each case that contained invalid values was manually inspected per phase using the raw data files (i.e., ECG data). The goal of this inspection was to determine whether there were at least 30 consecutive seconds (i.e. 3 datapoints) of sound data preceding or succeeding the invalid value(s) within the phase. When there were indeed at least 30 consecutive seconds (i.e. 3 data points) of sound data within the phase, these data points were used to calculate an average score of the specific phase, and the invalid values were deleted. When there were less than 30 consecutive seconds (i.e. 3 data points) of sound data within the phase, the phase was deleted from the dataset altogether. This resulted in the following number of cases that were used in the final dataset (Table A2).

**Table A2**

*Final number of cases in the dataset after deleting invalid values*

|  |  | N | Baseline | Mask | Spider | Robot | Recovery |
| --- | --- | --- | --- | --- | --- | --- | --- |
| HRV T1 |  | 37 |  |  |  |  |  |
|  | Final set |  | 33 | 32 | 35 | 34 | 35 |
|  | Adapted^1^ |  | 3 | 2 | 2 | 2 | 7 |
|  | Deleted^2^ |  | 4 | 5 | 2 | 3 | 2 |
| HRV T2 |  | 41 |  |  |  |  |  |
|  | Final set |  | 38 | 34 | 37 | 39 | 38 |
|  | Adapted^1^ |  | 7 | 1 | 3 | 2 | 8 |
|  | Deleted^2^ |  | 3 | 7 | 4 | 2 | 3 |
| HRV T3 |  | 45 |  |  |  |  |  |
|  | Final set |  | 44 | 42 | 42 | 41 | 43 |
|  | Adapted^1^ |  | 10 | 3 | 5 | 4 | 13 |
|  | Deleted^2^ |  | 1 | 3 | 3 | 4 | 2 |

*Note.* HRV = heart rate variability. ^1^Adapted = invalid value(s) were deleted within the phase, with the remaining minimum of 30 consecutive seconds used to calculate the average HRV of the specific phase. ^2^ Deleted = phase was deleted from the dataset, as there was no minimum of 30 consecutive seconds to calculate the average HRV with.
